# Factors Influencing COVID-19 Vaccination Intentions Among College Students: A Cross-Sectional Study in India

**DOI:** 10.3389/fpubh.2021.735902

**Published:** 2021-12-15

**Authors:** Lovely Jain, Jatina Vij, Prakasini Satapathy, Venkatesan Chakrapani, Binod Patro, Sitanshu Sekhar Kar, Ritesh Singh, Star Pala, Lalit Sankhe, Bhavesh Modi, Surya Bali, Neeti Rustagi, Vineeth Rajagopal, Tanvi Kiran, Kapil Goel, Arun Kumar Aggarwal, Madhu Gupta, Bijaya Kumar Padhi

**Affiliations:** ^1^Department of Community Medicine, School of Public Health, Post Graduate Institute of Medical Education & Research (PGIMER), Chandigarh, India; ^2^Department of Public Health, Utkal University, Bhubaneswar, India; ^3^Centre for Sexuality and Health Research and Policy (C-SHaRP), Chennai, India; ^4^Department of Community and Family Medicine, All India Institute of Medical Sciences (AIIMS), Bhubaneswar, India; ^5^Department of Preventive and Social Medicine, Jawaharlal Institute of Postgraduate Medical Education & Research (JIPMER), Puducherry, India; ^6^Department of Community and Family Medicine, All India Institute of Medical Sciences (AIIMS), Kalyani, India; ^7^Department of Community Medicine, North Eastern Indira Gandhi Regional Institute of Health & Medical Sciences (NEIGRIHMS), Shillong, India; ^8^Grant Medical College, JJ Hospital, Mumbai, India; ^9^Community Medicine Health & Family Welfare Department, Government of Gujarat, Gandhinagar, India; ^10^Department of Community and Family Medicine, All India Institute of Medical Sciences (AIIMS), Bhopal, India; ^11^Department of Community and Family Medicine, All India Institute of Medical Sciences (AIIMS), Medical Research Public University, Jodhpur, India

**Keywords:** COVID-19, students, vaccine hesitancy, vaccine uptake, vaccine intention, first wave

## Abstract

**Background:** Students act as messengers in delivering effective messages for better uptake of health-promoting behavior. Understanding their knowledge about coronavirus disease 2019 (COVID-19), intentions to use the COVID-19 vaccine, and its associated factors will help develop promising strategies in vaccine promotion concerning the current COVID-19 pandemic.

**Methods:** A cross-sectional online survey was carried out among students in the healthcare and non-healthcare sectors to assess their intentions to get vaccinated against the COVID-19. A non-probability snowball sampling technique was used to recruit study participants (*N* = 655) through social media platforms and emails. Study participants were recruited across the country, including six major geographical regions (Eastern, Western, Northern, Southern, North-east, and Central) in India between November 2020 and January 2021 before the introduction of the COVID-19 vaccine. Descriptive statistics were used to present the sociodemographic, and vaccine-related behaviors of the study participants. Key determinants that likely predict vaccine acceptance among students were modeled using logistic regression analysis. For each analysis, *p* < 0.05 was considered significant.

**Results:** A total of 655 students were recruited, 323 from healthcare and 332 from non-healthcare sectors, to assess their intentions to receive the COVID-19 vaccine. Of the 655 students, 63.8% expressed intentions to receive the COVID-19 vaccine. The acceptance was higher among non-healthcare students (54.07 vs. 45.93%). At the time of the study, 27.8% of the students indicated that they had been exposed to a confirmed COVID-19 patient. A vast majority (93.4%) of the students knew about the COVID-19 virus, and most (89.3%) of them were aware of the development of a COVID-19 vaccine. The history of vaccine hesitancy was found to be low (17.1%). Only one-third (33.4%) of the students showed concern about contracting COVID-19. Trust in the healthcare system [adjusted odds ratio (aOR): 4.13; (95% CI: 2.83–6.04), *p* < 0.00] and trust in domestic vaccines [aOR: 1.46; (95% CI: 1.02–2.08), *p* < 0.05] emerged as the significant predictors of student's intention to get vaccinated. Higher acceptance for vaccine was observed among students in the non-healthcare [aOR: 1.982; 95% CI: 1.334–2.946, *p* < 0.00].

**Conclusion:** This study shows that the Indian college students had relatively high levels of positive intentions to receive COVID-19 vaccines, although about one-third were not sure or unwilling to receive the vaccine, highlighting possible vaccine hesitancy. Informational campaigns and other strategies to address vaccine hesitancy are needed to promote uptake of COVID-19 vaccines.

## Introduction

The coronavirus disease 2019 (COVID-19) pandemic has continued to affect multiple domains of human lives since its emergence at theend of 2019 (1). The increasing cases of COVID-19 had an adverse effect on all spheres of life, the economy, and the environment during the lockdown period (2). On 30 January 2020, the World Health Organization (WHO) declared an outbreak, a Public Health Emergency of International Concern (3). The COVID-19 pandemic has significantly affected the lives of college students physically, academically, financially, and psychologically (4). To prevent widespread transmission of the COVID-19 virus among college staff and the young adult population, higher-education institutions across the country have rapidly switched from in-person to online learning (5). Growing attention has been paid to vaccinate high-risk populations to control the COVID-19 pandemic. Young adults and students are critical populations for the vaccination campaign and could act as messengers in delivering effective messages for better uptake of vaccines among their families and communities (6). Students in the healthcare sector are considered to have high-risk exposures to COVID-19 and have been given priority in vaccine allocations (6). In India, the COVID-19 vaccine was introduced in a phased manner with the first phase focused on healthcare workers, frontline workers, and populations at high risk as part of concerned control measures (7). Covishield (Serum Institute of India) and Covaxin (Bharat Biotech) were the two vaccines approved for restricted emergency use (8, 9). However, at the time of the study, acceptance of these vaccines remained skeptical, with safety (10) and efficacy (11) as major concerns. Students and younger populations seemed to play an important role in determining vaccine acceptance in the community, with contrasting effects in different countries (12), but information on the same was lacking in India.

Vaccine hesitancy was a significant issue even before the COVID-19 pandemic (13). The WHO considered this phenomenon as one of the top ten threats in global health in 2019. The available evidence seems to show that the intention to get vaccinated against COVID-19 is lower among younger adults and young people (14). Younger individuals may believe that COVID-19 poses a less serious threat to themselves than to other age groups (15). To attain complete protection from the COVID-19 viruses, the vaccines need to be widely accepted by all subgroups of the populations, including youth (16). In the United States of America (USA), though it reported the highest number of confirmed cases of COVID-19 in 2020, only half of the medical students expressed willingness to participate in a vaccine trial and about 23% were hesitant to get vaccination (17). Similarly, only 35% of the medical students in Egypt (18), 45% of nursing students in Europe (19), and 52.8% of college students in New Jersey, USA, indicated their intention to receive COVID-19 vaccine (4). Although, a majority (86.1%) of the university students in Italy were willing to receive COVID-19 vaccine (20). Numerous studies reported that lack of trust in healthcare and/or health officials are common barriers to vaccination (21–23). However, there is a lack of such information from India on the prevalence of vaccination intentions and the reasons for such intentions among students in India.

Given that students could play an essential role in the global vaccination campaign by influencing the vaccination intentions of their families and communities, their perception and acceptance of COVID-19 vaccines, including factors associated with those intentions, need to be investigated. Accordingly, we conducted a study to assess the intention to receive the COVID-19 vaccine and predictors of uptake among students in India. The findings from this study could help policymakers to prioritize strategies for effective interventions to promote vaccination among college students.

## Methods

### Study Design and Sample

A cross-sectional study was conducted among college students including healthcare and non-healthcare sectors across India. The survey was conducted between November 2020 and January 2021 during the “first wave” and before the introduction of COVID-19 vaccines in India. Students (*N* = 655) were recruited from an online survey *via* a self-reported questionnaire through social media, like WhatsApp, Facebook, and Twitter. Thus, primarily a convenient sampling technique was used. Further, the snowball sampling technique was used for enlisting additional participants, where the invited participants were requested to pass on the invitations to their peers. The minimum required sample size was estimated to be 576 with a 50% acceptance rate, 5% absolute precision, and 95% confidence level. The design effect was kept at 1.5. We recruited a total of 655 students of which 323 students were from healthcare and 332 students were from non-healthcare sectors. Participants aged 18 years or above who are currently living in India were included in the study. An online informed consent was obtained from all the participants before the survey. Only those provided the informed consent were taken to the questionnaire web page to initiate the survey.

### Study Questionnaire

The questionnaire was informed by a literature review of similar studies (24, 25). The survey assessed various domains, such as (1) previous immunization behaviors; (2) perception of vaccines; (3) current knowledge about COVID-19 and personal experiences about COVID-19; and (4) sociodemographic characteristics, including age, sex, marital status, educational status, family size and income, social status, religion, caste, and residence. Some of the key questions that were asked include whether “they ever refused a vaccine for themselves or a child considering it as useless or dangerous”; “if they ever postponed a vaccine recommended by the physician” for assessing the general perception toward vaccine; “before the interview if they were aware of the COVID-19 virus that is currently circulating in the community”; “Is there currently any vaccine being prepared for the pandemic Coronavirus strain”; “do you have any history of staying or traveling with confirmed COVID-19 patient”; and “how concern are you that your family member will be infected with COVID-19 virus,” for estimating the current knowledge and personal experience about COVID-19.

### Statistical Analysis

Descriptive analysis was conducted for socio-demographic characteristics of the responses to the survey. The age variable was categorized into three groups: 18–25, 26–35, and above 35 years. Gender was coded as a binary variable (male and female). Marital status was grouped into two categories (married and single). The educational status was categorized into three groups (postgraduate, graduate, and high school/diploma). Five response options were provided to family monthly income < INR 5,000 (about USD 71), 5,000–10,000, 11,000–20,000, 21,000–50,000, and >50,000. The geographical region of India was grouped into six categories (East, West, North, South, Central, and North-east), and the place of residence was categorized into urban vs. rural. “Yes” and “no” options were used for categorizing responses to certain questions, such as previous immunization behavior, perception of vaccines, awareness about COVID-19, and personal experience concerning COVID-19. In addition, they were asked “How concerned you are that you or someone in your family will be infected with COVID-19 virus?” and the responses were recorded in a three-point Likert scale (very concerned, somewhat concerned, and not at all concerned). “Do you have trust in the healthcare system to manage the current situation related to COVID-19?” was categorized into very likely, somewhat likely, and not at all. Bivariate analysis was conducted between all variables with the dependent variable of interest. Multiple logistic regression analysis was conducted to examine the association between the students receiving the COVID-19 vaccine with their sociodemographic and vaccination behaviors. A *p* < 0.05 was considered significant. Data analysis was conducted using Stata 15 software (STATA Corporation, College Station, TX, USA).

### Ethical Consideration

The study was approved by the Institutional Research Ethics Committee of the Postgraduate Institute of Medical Education and Research (PGIMER), Chandigarh, India. All participants provided informed consent. Steps were taken to ensure confidentiality and privacy of the information provided by the participants, and only de-identified data were used.

## Result

A total of 655 students participated in the study, with 49.3% from healthcare sectors and 50.6% from non-healthcare sectors. Of these 655 students, 42.8 and 22.4% belonged to the Northern and Eastern parts of India, respectively. Around 43.2% were graduates, and 41.1% were postgraduate students. About half of the students were in the age group of 18–25 years (54.0%). About three-fifths (61.9%) were women and 68.5% were unmarried. A majority (79.7%) of students belonged to middle socioeconomic status, with (41.0%) reporting a family monthly income of >INR 50,000 (about USD 710) (Table 1).

**Table 1:** Socio-demographic characteristics of the study participants (*N* = 655).

**Variables**	***n*** **(%)**
**Age (in years)**	
18–25	390 (59.54%)
26–35	242 (36.95%)
above 35	23 (3.51%)
**Gender**	
Male	249 (38.02%)
Female	406 (61.98%)
**Students' category**	
Healthcare sector	323 (49.31%)
Non-healthcare sector	332 (50.69%)
**Highest education**	
Diploma/higher secondary school	99 (15.11%)
Undergraduate	287 (43.82%)
Postgraduate	269 (41.07%)
**Marital status**	
Married	198 (30.23%)
Single	457 (69.77%)
**Family size**	
Five and below	517 (78.93%)
Six and above	138 (21.07%)
**Family income (INR/month)**	
below 10,000	79 (12.06%)
11,000–20,000	110 (16.79%)
21,000–50,000	198 (30.23%)
above 50,000	268 (40.92%)
Social status in the community*	
Low	66 (10.08%)
Medium	522 (79.69%)
High	67 (10.23%)
**Geographical region in India**	
Eastern	147 (22.44%)
Western	55 (8.40%)
Northern	280 (42.75%)
Southern	50 (7.63%)
Central	60 (9.16%)
North-east	63 (9.62%)
**Place of residence**	
Urban	309 (47.18%)
Rural	346 (52.82%)

* *Self-reported information about perceived social status in participants neighborhood*.

Most (93.4%) of the participants were aware that the COVID-19 virus was circulating in the community; 89.3% of the students were aware that a “COVID-19 vaccine” was being prepared. About a little less than two-thirds (64.5%) of the students reported having trust in the healthcare system of India, and a little more than half (56.0%) reported having confidence in domestic vaccines. About 27.7% of students reported having had a history of travel or contact with a confirmed COVID-19 patient. Only one-third (33.4%) of the students were concerned about contracting the COVID-19 virus. A large proportion of students (82.9%) did not have any history of vaccine hesitancy (Table 2). A majority (89.3%) of the students knew that COVID-19 vaccines were being developed, had trust in the healthcare system (64.5%) and domestic vaccines (56.0%). Only about one-third (33.4%) perceived themselves at risk of contracting Coronavirus infection (Table 2).

**Table 2:** Descriptive statistics on knowledge, risk perception, trust on the healthcare system, and perception toward domestic COVID-19 vaccine among the study participants (*N* = 655).

**Variables**	***n*** **(%)**
**Exposed to COVID-19 cases**	
No	473 (72.21%)
Yes	182 (27.79%)
**Awareness about COVID-19**	
No/not sure	43 (6.56%)
Yes	612 (93.44%)
**Awareness about development of COVID-19 vaccines**	
No/not sure	70 (10.69%)
Yes	585 (89.31%)
**History of vaccine hesitancy**	
Yes	112 (17.10%)
No	543 (82.90%)
**Risk perception**	
Yes	219 (33.44%)
No	436 (66.56%)
**Trust in the healthcare system**		
No	232 (35.42%)
Yes	423 (64.58%)
**Trust in domestic vaccines**		
No	288 (43.97%)
Yes	367 (56.03%)

In bivariate analysis, intention to receive COVID-19 vaccine seems to be slightly higher among non-healthcare sector students than among healthcare sector students (68.1 vs. 59.4%, *p* = 0.02), higher among unmarried students when compared to those who were single (33.9 vs. 66.0%), and higher among postgraduate (42.8%) and undergraduate students (40.1%) when compared to diploma/higher secondary students (Table 3). Knowledge about the development of the COVID-19 vaccine, risk perception, trust in the healthcare system, and trust in the domestic vaccine was found to be independently associated with vaccine intention among students (Table 4).

**Table 3:** Bivariate analysis between sociodemographics and intention to receive COVID-19 vaccines among the study participants (*N* = 655).

**Variables**	**Intention to receive COVID-19 vaccines**		***P* value**
	**No/not sure (*n* = 237)**	**Yes (*n* = 418)**	
**Age (in years)**			0.06
18–25	154 (64.98%)	236 (56.46%)	
26–35	78 (32.91%)	164 (39.23%)	
above 35	5 (2.11%)	18 (4.31%)	
**Gender**			0.25
Male	97 (40.93%)	152 (36.36%)	
Female	140 (59.07%)	266 (63.64%)	
**Students' category**			0.02
Healthcare sector	131 (55.27%)	192 (45.93%)	
Non-healthcare sector	106 (44.73%)	226 (54.07%)	
**Marital status**			0.01
Married	56 (23.63%)	142 (33.97%)	
Single	181 (76.37%)	276 (66.03%)	
**Highest education**			0.03
Diploma/higher secondary school	28 (11.81%)	71 (16.99%)	
Undergraduate	119 (50.21%)	168 (40.19%)	
Postgraduate	90 (37.97%)	179 (42.82%)	
**Social status in the community**			0.68
Low	24 (10.13%)	42 (10.05%)	
Medium	192 (81.01%)	330 (78.95%)	
High	21 (8.86%)	46 (11.00%)	
**Family income (INR/month)**			0.72
below 10,000	31 (13.08%)	48 (11.48%)	
11,000–20,000	39 (16.46%)	71 (16.99%)	
21000–50,000	76 (32.07%)	122 (29.19%)	
above 50000	91 (38.40%)	177 (42.34%)	
**Family size**			0.31
Five and below	182 (76.79%)	335 (80.14%)	
Six and above	55 (23.21%)	83 (19.86%)	
**Place of residence**			0.60
Urban	115 (48.52%)	194 (46.41%)	
Rural	122 (51.48%)	224 (53.59%)	
**Geographical region in India**			0.06
Eastern	48 (20.25%)	99 (23.68%)	
Western	16 (6.75%)	39 (9.33%)	
Northern	117 (49.37%)	163 (39.00%)	
Southern	20 (8.44%)	30 (7.18%)	
Central	14 (5.91%)	46 (11.00%)	
North-east	22 (9.28%)	41 (9.81%)	

**Table 4:** Bivariate analysis between vaccine behavior, risk perception, and intention to receive COVID-19 vaccines among the study participants (*N* = 655).

**Variables**	**“Intended to uptake COVID-19 vaccine”**		***P*** **value**
	**No/not sure (***n*** = 237)**	**Yes (***n*** = 418)**	
**Exposed to COVID-19 cases**			0.74
No	173 (73.00%)	300 (71.77%)	
Yes	64 (27.00%)	118 (28.23%)	
**Awareness about COVID-19**			0.14
No/not sure	20 (8.44%)	23 (5.50%)	
Yes	217 (91.56%)	395 (94.50%)	
**Awareness about development of COVID-19 vaccines**			0.00
No/not sure	36 (15.19%)	34 (8.13%)	
Yes	201 (84.81%)	384 (91.87%)	
**History of vaccine hesitancy**			0.33
Yes	36 (15.19%)	76 (18.18%)	
No	201 (84.81%)	342 (81.82%)	
**Risk perception**			0.03
Yes	92 (38.82%)	127 (30.38%)	
No	145 (61.18%)	291 (69.62%)	
**Trust in the healthcare system**			0.00
No	131 (55.27%)	101 (24.16%)	
Yes	106 (44.73%)	317 (75.84%)	
**Trust in domestic vaccines**			0.00
No	126 (53.16%)	162 (38.76%)	
Yes	111 (46.84%)	256 (61.24%)	

Table 5 shows a multivariable logistic regression analysis of the factors influencing the COVID-19 vaccine uptake among students. We found that those who trusted the healthcare system (vs. those who did not trust) (aOR = 4.13, 95% CI: 2.83–6.04, *p* < 0.001), and those who had confidence in domestic vaccines (vs. those who did not have confidence) (aOR = 1.46, 95 % CI: 1.02–2.08, *p* = 0.03) had higher odds of reporting willingness to receive COVID-19 vaccines.

**Table 5:** Multivariable logistic regression analysis of the influencing factors on the COVID-19 vaccine uptake among study participants (*N* = 655).

**Variables**	**“Intended to uptake COVID-19 vaccine”**	
	**aOR [95% CI]**	***P*** **value**
**Trust in the healthcare system**		
No	Ref	
Yes	4.13 [2.83, 6.04]	<0.001
**Trust in domestic vaccines**		
No		
Yes	1.46 [1.02, 2.08]	0.038
**Exposed to COVID-19 cases**		
No	Ref	
Yes	0.96 [0.65, 1.42]	0.873
**Risk perception**		
No		
Yes	1.30 [0.89, 1.89]	0.165
**History of vaccine hesitancy**		
Yes	Ref	
No	0.85 [0.52, 1.37]	0.518
**Age (in years)**		
18–25	Ref	
26–35	1.11 [0.74, 1.67]	0.604
above 35	1.60 [0.53, 4.81]	0.397
**Gender**		
Male	Ref	
Female	1.25 [0.87, 1.81]	0.216
**Students' category**		
Healthcare sector	Ref	
Non-healthcare sector	1.98 [1.33, 2.94]	<0.01
**Marital status**		
Married	Ref	
Single	0.81 [0.52, 1.27]	0.367
**Highest education**		
Diploma/high school	Ref	
Undergraduate	0.59 [0.34, 1.02]	0.05
Postgraduate	0.73 [0.42, 1.28]	0.277
**Social status in the community**		
High		
Low	1.11 [0.50, 2.46]	0.780
Medium	0.99 [0.54, 1.82]	0.984
**Place of residence**		
Urban	Ref	
Rural	0.98 [0.69, 1.40]	0.951

## Discussion

Studies on COVID-19 in India have described the effect of COVID-19 on medical education (26), the relationship between COVID-19 and the suicidal tendency among healthcare professionals and students (27, 28) and COVID-19-related anxiety among students (29, 30). However, none have reported uptake of COVID-19 vaccine among students, especially those in the healthcare sector. This study is one of the first studies that examined the willingness of hypothetical vaccine for COVID-19 among students in India.

Our findings indicate a relatively good level of willingness (63.8%) among the healthcare and non-healthcare sectors to receive COVID-19 vaccines. However, the low or lack of intention to vaccinate among one-third of the sample in this study indicate vaccine hesitancy that needs to be tackled. Our findings are in line with the percentages of students (35–52.8%) who reported willingness to receive the COVID-19 vaccine in European countries and the USA (4, 18). In this study, we also found that the students in the non-healthcare sector were more willing to receive COVID-19 vaccines (54.0%) than those in the healthcare sector (45.9%). We also found that trust in the healthcare system and domestic vaccines was related to intention to receive COVID-19 vaccines. This finding of ours is similar to a survey conducted among college students in South Carolina, where 73.9% of students trust healthcare authorities, and that trust was significantly associated with COVID-19 vaccine acceptance (31). While this study found high levels of trust in domestic vaccines and their association with COVID-19 vaccine acceptance, a study conducted in China reported that imported vaccine was preferred over domestic vaccine (32). Concerning the risk perception for COVID-19 infection, in this study, the participants reported a lower risk perception of getting infected, possibly reflecting an optimism bias, our expectations (low risk of infection) are better than reality (actual risk of infection) (15, 33).

In multivariable analysis, age, gender, and education were not significantly associated with vaccine acceptance. This finding contrasts with the results of other studies conducted in Portugal (23) and Indonesia (34), where vaccine acceptance was significantly associated with education and marital status, respectively; a lower intention to be vaccinated against COVID-19 among women was also reported (35). Perceived adverse events of the COVID-19 vaccine could be a possible reason for hesitancy, as highlighted by various studies among university students and the general population (18, 31). Studies have shown that adverse events and poor vaccine efficacy could be possible reasons for vaccination hesitancy among medical students (36). Studies among other populations too have reported several reasons for hesitancy toward COVID-19 vaccines, including side-effects of the vaccine (37), speed of development of the vaccine (38), uncertainty about the effectiveness (39), and effective duration of the vaccine, and medical mistrust (40).

Students are considered as trusted influencers and ambassadors for vaccine promotion (41). Vaccine hesitancy among students, especially those in the healthcare sector, and other priority groups has potentially negative consequences to themselves and may influence vaccine acceptance among the general population (42). For example, one study reported that vaccinated healthcare providers were more likely to recommend vaccination to their patients (41).

This study has several limitations. First, being a cross-sectional study, the predictors of vaccine acceptance may not be causal and may only reflect potential associations through unknown mechanisms. However, the identified significant predictors are in line with the potential mechanisms of vaccine acceptance documented in the literature. Nevertheless, the intention to receive COVID-19 vaccines may vary over time and structural contexts. Second, the study used convenient and snowball sampling to recruit participants, and thus the results may not be generalizable to all students of healthcare and non-healthcare sectors. Third, an online self-administered questionnaire form was used instead of a face-to-face interview, with potential bias in reporting; however, studies in the health field have shown that there may not be a difference between a self-administered and interviewer-administered questionnaire (43, 44). The reasons for the refusal or low willingness to receive COVID-19 vaccines need further investigation, possibly using qualitative or mixed methods approaches. Despite these limitations, this study provides critical information on the factors that impact the acceptance of COVID-19 vaccination among college students in India. Furthermore, the data presented in this study could act as a piece of baseline information for future comparisons, i.e., how the intention to vaccinate varied over time.

## Conclusion

In this study, we found suboptimal levels of willingness to receive COVID-19 vaccines, with nearly one-third not sure or willing to receive a vaccine, indicating high levels of potential vaccine hesitancy. Trust in the healthcare system, including vaccine safety and effectiveness, was a significant factor for COVID-19 vaccine acceptance among students. It is pertinent to design an evidence-based strategy to promote the uptake of vaccination among students, which could include informational campaigns that address vaccine hesitancy. In addition, strategies demonstrating the effectiveness and safety of the vaccine will be essential when rolling out the COVID-19 vaccination programs and those strategies will certainly have implications beyond the COVID-19 pandemic.

## Data Availability Statement

The original contributions presented in the study are included in the article/supplementary material, further inquiries can be directed to the corresponding author.

## Ethics Statement

The studies involving human participants were reviewed and approved by Institutional Research Ethics Committee of Post Graduate Institute of Medical Education & Research (PGIMER), Chandigarh, India. The patients/participants provided their written informed consent to participate in this study.

## Author Contributions

BP, LJ, MG, and AA conceptualized the study and designed the tools. BP, LJ, JV, PS, VC, BP, SK, RS, SP, SB, NR, VR, TK, KG, BM, LS, MG, and AA conducted the study at the national level and collected the data. BP and LJ analyzed the data. LJ drafted the introduction. BP drafted the methods and results. LJ and VC drafted the discussion. MG and AA provided guidance, particularly related to the strategy and recommendations. All authors reviewed drafts, provided edits, and approved the final submission.

## Conflict of Interest

The authors declare that the research was conducted in the absence of any commercial or financial relationships that could be construed as a potential conflict of interest.

## Publisher's Note

All claims expressed in this article are solely those of the authors and do not necessarily represent those of their affiliated organizations, or those of the publisher, the editors and the reviewers. Any product that may be evaluated in this article, or claim that may be made by its manufacturer, is not guaranteed or endorsed by the publisher.

## Abbreviations

COVID-19: Coronavirus disease-2019
WHO: World Health Organization.
